# Genotype-based “virtual” metabolomics in a clinical biobank identifies novel metabolite-disease associations

**DOI:** 10.3389/fgene.2024.1392622

**Published:** 2024-05-15

**Authors:** Minoo Bagheri, Andrei Bombin, Mingjian Shi, Venkatesh L. Murthy, Ravi Shah, Jonathan D. Mosley, Jane F. Ferguson

**Affiliations:** ^1^ Division of Cardiovascular Medicine, Department of Medicine, Vanderbilt University Medical Center, Nashville, TN, United States; ^2^ Department of Biomedical Informatics, Vanderbilt University Medical Center, Nashville, TN, United States; ^3^ Division of Cardiovascular Medicine, University of Michigan, Ann Arbor, MI, United States; ^4^ Division of Clinical Pharmacology, Department of Medicine, Vanderbilt University Medical Center, Nashville, TN, United States

**Keywords:** Mendelian randomization, metabolite, phenotype, polygenic score, virtual metabolomics

## Abstract

**Introduction:** Circulating metabolites act as biomarkers of dysregulated metabolism and may inform disease pathophysiology. A portion of the inter-individual variability in circulating metabolites is influenced by common genetic variation. We evaluated whether a genetics-based “virtual” metabolomics approach can identify novel metabolite-disease associations.

**Methods:** We examined the association between polygenic scores for 724 metabolites with 1,247 clinical phenotypes in the BioVU DNA biobank, comprising 57,735 European ancestry and 15,754 African ancestry participants. We applied Mendelian randomization (MR) to probe significant relationships and validated significant MR associations using independent GWAS of candidate phenotypes.

**Results and Discussion:** We found significant associations between 336 metabolites and 168 phenotypes in European ancestry and 107 metabolites and 56 phenotypes in African ancestry. Of these metabolite-disease pairs, MR analyses confirmed associations between 73 metabolites and 53 phenotypes in European ancestry. Of 22 metabolitephenotype pairs evaluated for replication in independent GWAS, 16 were significant (false discovery rate p < 0.05). These included associations between bilirubin and X–21796 with cholelithiasis, phosphatidylcholine (16:0/22:5n3,18:1/20:4) and arachidonate with inflammatory bowel disease and Crohn’s disease, and campesterol with coronary artery disease and myocardial infarction. These associations may represent biomarkers or potentially targetable mediators of disease risk.

## Introduction

Dysregulated metabolism underlies many of the leading causes of morbidity and mortality, causing considerable human suffering, and high healthcare costs (American Diabetes Association, 2013; Mozaffarian et al., 2016; National Diabetes Statistics Report, 2017). The adverse clinical consequences of extreme disruptions of metabolite homeostasis caused by inborn errors of metabolism are well recognized (Mootha and Hirschhorn, 2010). However, modest, long-term perturbations of metabolites attributable to common genetic variation also may contribute to disease risk. The clinical consequences of these perturbations remains incompletely defined. Many complex diseases have residual risk that is not explained by our current knowledge of disease biology and mechanisms (Lieb et al., 2018). Identifying associations between circulating metabolites and diseases has the potential to identify biomarkers that can be used to risk-stratify individuals, and provide insight into disease mechanisms and enable targeted therapies.

Genome wide association studies (GWAS) for circulating metabolites measured by broad metabolomic profiling have identified numerous associated single nucleotide polymorphisms (SNPs) (Rhee et al., 2013; Shin et al., 2014; Demirkan et al., 2015; Kettunen et al., 2016; Rhee et al., 2016). These data can be repurposed to develop genetic instruments of individual metabolite levels which can be used to test for associations between metabolites and disease (Davey Smith and Ebrahim, 2003; Maher, 2015; Pasaniuc and Price, 2017). High throughput methodologies, such as Phenome-Wide Association Studies (PheWAS), test associations between genetic instruments and large number of clinical phenotypes using Electronic Health Record (EHR)-linked DNA biobanks (Denny et al., 2010; Karnes et al., 2017). These approaches can have significant advantages over traditional epidemiological approaches, allowing for highly-powered analyses which would otherwise be unfeasible due to cost or logistics. In this context, a ‘virtual’ metabolomics approach provides a powerful tool to identify candidate disease pathways, and to advance risk prediction beyond standard genetic models.

To define the broader phenome associated with circulating metabolites, we applied a virtual metabolomics approach that leveraged a large collection of clinical phenotypes derived from Vanderbilt’s BioVU EHR-linked DNA biobank. We constructed virtual metabolomes based on metabolite polygenic scores (PGS), to identify clinical diagnoses that shared genetic modulators with metabolites. Mendelian randomization approaches were then used to better define the relationship between candidate metabolite-phenotype pairs. Significant associations were further validated using external data sets. Our data shed light on multiple metabolite-disease relationships and highlight novel pathways for potential therapeutic intervention.

## Material and methods

### Vanderbilt BioVU study population

Genetic and phenotypic data were obtained from BioVU, Vanderbilt University Medical Center’s (VUMC) DNA Biobank linked to a de-identified electronic health record (Roden et al., 2008). The study population comprised individuals of genetic white European (n = 57,735) and African (n = 15,754) ancestries, 18 years and older who had existing SNP genotyping. Genetic ancestry of individuals was determined using principal component analysis in conjunction with HAPMAP reference sets (Gibbs et al., 2003; Roden et al., 2008). This study was reviewed by the VUMC Institutional Review Board (IRB) in accordance with the informed consent guidelines and was determined to be non-human subjects research.

### Genetic data and quality control

BioVU participants were genotyped on the Illumina Infinium Multi-Ethnic Genotyping Array (MEGA^EX^) platform. Quality control procedures for this population have been described previously (Ruderfer et al., 2019). Individuals with a biological sex discrepancy or who were related (one participant from each related pair [pi-hat > 0.2] was randomly excluded) were excluded. Analyses used PLINK v1.9 (Purcell et al., 2007). Genotype imputation was performed using IMPUTE4 (Howie et al., 2009) version 2.3.0 (University of Oxford), using the 10/2014 release of the 1,000 Genomes cosmopolitan reference haplotypes. Genetic variants with imputation quality scores less than 0.3 were excluded. Principal components (PCs) to adjust for residual population stratification were generated using SmartPCA (Price et al., 2006).

### Phenotype data

For the BioVU population, the primary analyses examined clinical diagnoses based on PheCodes (v1.2), which are derived from International Classification of Disease (ICD) billing codes (ICD-9-CM and ICD-10 diagnosis codes) (Denny et al., 2010; Denny et al., 2013). Validated EHR algorithms were used to define phenotypes.^46^ For each phenotype, cases were defined as participants with at least two PheCode instances in their medical record. Individuals without any closely related PheWAS codes and who fell within the observed age of the cases were used as controls. We analyzed associations for 1,247 and 600 PheCodes with ≥100 cases in the European and African ancestry population, respectively.

### Specification of a virtual metabolome via human genetics

Discovery: Validated PGSs for 724 metabolites were obtained from the OMICSPRED resource (www.omicspred.org). (Xu et al., 2022) These PGS were developed using SNPs that significantly (*p* < 5 × 10^−8^) associated with concentrations of human blood metabolites in the INTERVAL cohort (n = 8,153 healthy individuals in England) (Xu et al., 2023). Briefly, metabolites were measured in plasma by an untargeted mass spectrometry metabolomics platform (Metabolon HD4), and participants were genotyped using the Affymetrix Biobank Axiom array (Shin et al., 2014). Bayesian ridge regression was used to develop genetic scores for each metabolite, and scores were validated (Spearman correlation) using an independent validation INTERVAL subset (n = 8,114 non-overlapping participants, 527 validated metabolites) and an external validation cohort (ORCADES, n = 1,007 European participants, 455 validated metabolites).

Validation: SNP instruments used for validation of predicted metabolite-disease associations by Mendelian randomization (MR) analyses were derived from the independent METSIM Finnish population study using publicly available GWAS summary statistics for metabolites (Yin et al., 2022). This study included 1,391 metabolites quantified in 6,136 non-diabetic male participants of Finnish ancestry. Summary statistics were obtained from the METSIM Metabolomics PheWeb server (https://pheweb.org/metsim-metab).

### Polygenic score analysis

SNPs associated with each of the 724 OMICSPRED metabolites were used to calculate PGSs as a weighted sum of trait-associated alleles for BioVU subjects described above, with PLINK v2.00a3LM (Purcell et al., 2007). Briefly, to construct PGS, we obtained SNPs related to each metabolite (*p* < 5 × 10^−8^) according to the OMICSPRED data, and used PLINK2 to compute polygenic scores using a list of SNPs and their scores (coefficient). Of the 724 metabolites, 102 had PGS that had no overlapping SNPs with other metabolites, while 622 comprised at least one SNP that was also part of the PGS for another metabolite. The association between metabolite PGS and each PheCode phenotype was tested using a multivariable logistic regression model, adjusting for sex and age. All analyses were stratified by genetic ancestry. Within each phenotype, association *p*-values were adjusted for multiple testing using a Benjamini–Hochberg false discovery rate (FDR) correction, (rstatix v0.7.0 R package).

### Mendelian randomization analysis to validate PGS associations

Phenotype and metabolite pairs that were significantly associated (FDR *p* < 0.05) with PGS through PheWAS in BioVU, were selected for MR analysis. MR tests for associations under three assumptions: (1) the SNPs are associated with the exposure; (2) the SNPs are not associated with confounders; and (3) the SNPs affect the outcome only through the exposure (Emdin et al., 2017). We used metabolite GWAS data from the independent METSIM study. Genetic instruments for each metabolite were selected based on suggestive significant associations (p < 5 × 10^−6^) in METSIM. We selected the p < 5 × 10^−6^ threshold, rather than a standard p < 5 × 10^−8^ threshold, as a pragmatic strategy to increase the number of SNPs included in the MR analysis. This allowed for greater inclusion of SNPs with potential biological relevance, but may decrease power or increase the chances of horizontal pleiotropy. We considered that the benefits of more expansive instruments outweighed these risks within the context of our robust multi-stage validation strategy. We applied a clumping algorithm to select an LD-reduced (*r* ^2^ < 0.05 with *physical distance* threshold of 1,000 kb) set of SNPs associated with metabolites. This resulted in 85,723 unique SNPs in European ancestry and 31,897 SNPs in the African ancestry population being included in the exposure instrumental variables. The association between metabolite-associated SNPs and the BioVU clinical phenotype of interest was computed using an additive logistic regression genetic model that adjusted for age, sex and 10 principal components (PLINK v2.00a3LM software). The inverse-variance weighted (random-effects the inverse-variance weighted (IVW)), MR-Egger (corrected for pleiotropy by setting the intercept to be non-zero) and weighted median (providing a consistent estimate of the causal effect with 50% of the information coming from valid instrument) methods, (Bowden et al., 2016), as implemented in the MendelianRandomization R package (Mahajan et al., 2018) were used to perform the analyses. Horizontal pleiotropy was determined by a low heterogeneity *p*-value (*p* < 0.05) based on the Cochran’s Q statistic. *p-*values were adjusted for multiple testing using a Benjamini–Hochberg FDR correction, per tested phenotype. For non-pleiotropic associations (heterogeneity *p* > 0.05), we selected significant (FDR *p* < 0.05) metabolite-phenotype pairs based on the IVW model, that showed consistent findings across the other MR methods. For associations with evidence of pleiotropy, we used *MR-PRESSO* to identify and evaluate the contributions of pleiotropic SNPs. The MR-PRESSO workflow consistent of three analyses: 1) a global test which assessed the existence of horizontal pleiotropic variants and a p_global-test_<0.05 was considered suggestive of pleiotropic effects; 2) an outlier test which identified pleiotropic variants, and SNPs with a *p* < 0.05 were identified as outliers; and 3) a distortion test which compared causal estimates pre and post removal of outlying variants, and a *p* < 0.05 was considered indicative that association estimates were biased due to outlying SNPs (Zhu, 2021).

### MR validation in independent disease-specific GWAS datasets

We validated significant MR associations using summary statistics from published GWAS datasets, where available. Because MR that uses only 1 or 2 SNPs may be driven by pleiotropy that cannot be easily detected, we selected only metabolites with instruments comprising three or more independent SNPs (p < 5 × 10^−6^ and LD *r* ^2^ < 0.05) for independent validation. We further excluded associations with horizontal pleiotropy which was identified by a low *p*-value (*p* < 0.05) in the Cochran’s Q statistic. GWAS summary statistics for Inflammatory Bowel Disease (IBD) and Crohn’s disease were obtained from a meta-analysis of 59,957 individuals of European ancestry (de Lange et al., 2017). Summary statistics for cholelithiasis were obtained from FinnGen (19,023 cases, 195,144 controls; FinnGen Consortium Release 5) and UK Biobank (11,632 cases, 289,159 controls) (https://ctg.cncr.nl/software/summary_statistics) (Mi et al., 2022) For Atopic dermatitis, GWAS summary statistics were obtained from a multi-ancestry GWAS of 21,399 cases and 95,464 controls from populations of European, African, Japanese and Latino ancestries (Paternoster et al., 2015). Summary statistics for AD were obtained from a meta-analysis of 1,126,563 individuals of European ancestry (Wightman et al., 2021). GWAS summary statistics for CAD and myocardial infarction (MI) were downloaded from www.cardiogramplusc4d.org (Nikpay et al., 2015) which included a GWAS meta-analysis of ∼185,000 CAD cases and controls with a subgroup analysis in cases with a reported history of myocardial infarction (around 70% of the total number of cases). Summary statistics for neutrophil counts were obtained from a trans-ethnic GWAS meta-analyses of 746,667 participants, including 184,535 non-European individuals (Chen et al., 2020). High-density lipoprotein (HDL), low-density lipoprotein (LDL), total cholesterol and triglycerides (TG) were obtained from the Global lipids consortium phenotypes (http://lipidgenetics.org/) (Willer et al., 2013) including 188,577 European, East Asian, South Asian and African ancestry individuals. All statistical tests were two-sided and analyses used R v.4.0.2. The circlize package was used to create the circular plots.

## Results

### Predicted circulating levels of metabolites associate with a broad range of clinical phenotypes

**FIGURE 1:**
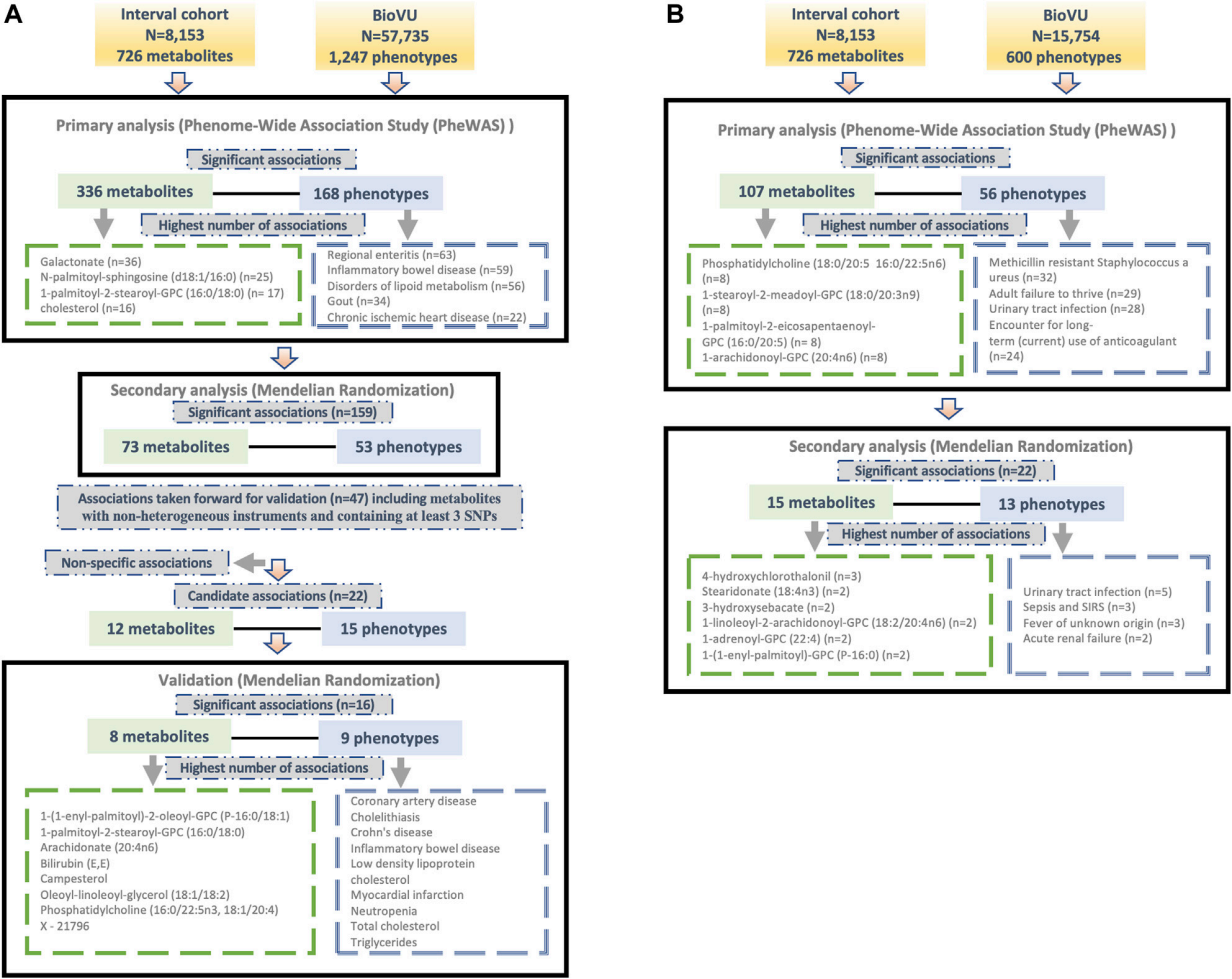
Overview of the study design and findings in **(A)** European and **(B)** African ancestry BioVU participants.

We tested for associations among PGS for 724 metabolites and up to 1,247 clinical phenotypes in BioVU. There were 336 metabolites significantly associated with 168 phenotypes in European ancestry (Supplementary Table S1) and 107 metabolites that were significantly (FDR *p* < 0.05) associated with 56 phenotypes in the African ancestry individuals (Supplementary Table S2). 78 metabolites, 11 phenotypes and 104 associations overlapped between European and African ancestry individuals. Clinical phenotypes with the highest number of significant metabolite associations included regional enteritis (n = 63), inflammatory bowel disease (n = 59), disorders of lipid metabolism (n = 56), gout (n = 34), and chronic ischemic heart disease (n = 22) in the European ancestry population [Figure 1A]. Within African ancestry, there were multiple associations between metabolites and methicillin resistant *Staphylococcus aureus* (n = 32; one amino acid, one unknown metabolite and 30 lipids), adult failure to thrive (n = 29), and urinary tract infection (n = 28) [Figure 1B].

Metabolites with the highest number of significant associations with phenotypes in European ancestry included galactonate (n = 36), N-palmitoyl-sphingosine (d18:1/16:0) (n = 25), 1-palmitoyl-2-stearoyl-GPC (16:0/18:0) (n = 17), and cholesterol (n = 16) [Figure 1A]. In African ancestry, phosphatidylcholine (18:0/20:5 16:0/22:5n6) (n = 8), 1-stearoyl-2-meadoyl-GPC (18:0/20:3n9) (n = 8), 1-palmitoyl-2-eicosapentaenoyl-GPC (16:0/20:5) (n = 8), 1-arachidonoyl-GPC (20:4n6) (n = 8), and 1-palmitoyl-2-arachidonoyl-GPC (16:0/20:4) (n = 7) associated with multiple phenotypes [Figure 1B].

### Mendelian randomization highlights relationships between circulating lipids and multiple disease phenotypes

For significant metabolite and phenotype pairs from PheWAS of metabolite PGS, we further characterized the associations under a MR framework. In European ancestry, of the 336 significant metabolites, GWAS summary statistics were available for 280 matched metabolites in the METSIM study. Of the study metabolites with no corresponding match in METSIM, 45 of 56 were unknown/unidentified metabolites. We identified 159 significant associations (FDR<0.05) among 73 metabolites and 53 phenotypes by IVW method (Figure 1A; Supplementary Table S3). Among these associations were several distinct phenotype groups with a high number of significant associations with metabolites including those related to dyslipidemia (hyperlipidemia [n = 13]; disorders of lipid metabolism [n = 11]; hyperglyceridemia [n = 8]; hypercholesterolemia [n = 8]), gastrointestinal disorders (inflammatory bowel disease [n = 8]; regional enteritis [n = 7]), metabolic disorders (disorders of bilirubin excretion [n = 8]; cholelithiasis and cholecystitis [n = 6], gout and other crystal arthropathies [n = 5]), decreased white blood cell count (n = 5), and nasal polyps (n = 2). The corresponding metabolites were predominately lipids, including 1-palmitoyl-2-palmitoleoyl-GPC (16:0/16:1) (n = 9), palmitoyl-linoleoyl-glycerol (16:0/18:2) (n = 8), palmitoyl sphingomyelin (d18:1/16:0) (n = 8), campesterol (n = 7), cholesterol (n = 6), 2-hydroxybutyrate/2-hydroxyisobutyrate (n = 5) and 1-(1-enyl-palmitoyl)-2-linoleoyl-GPC (P-16:0/18:2) (n = 5).

**FIGURE 2:**
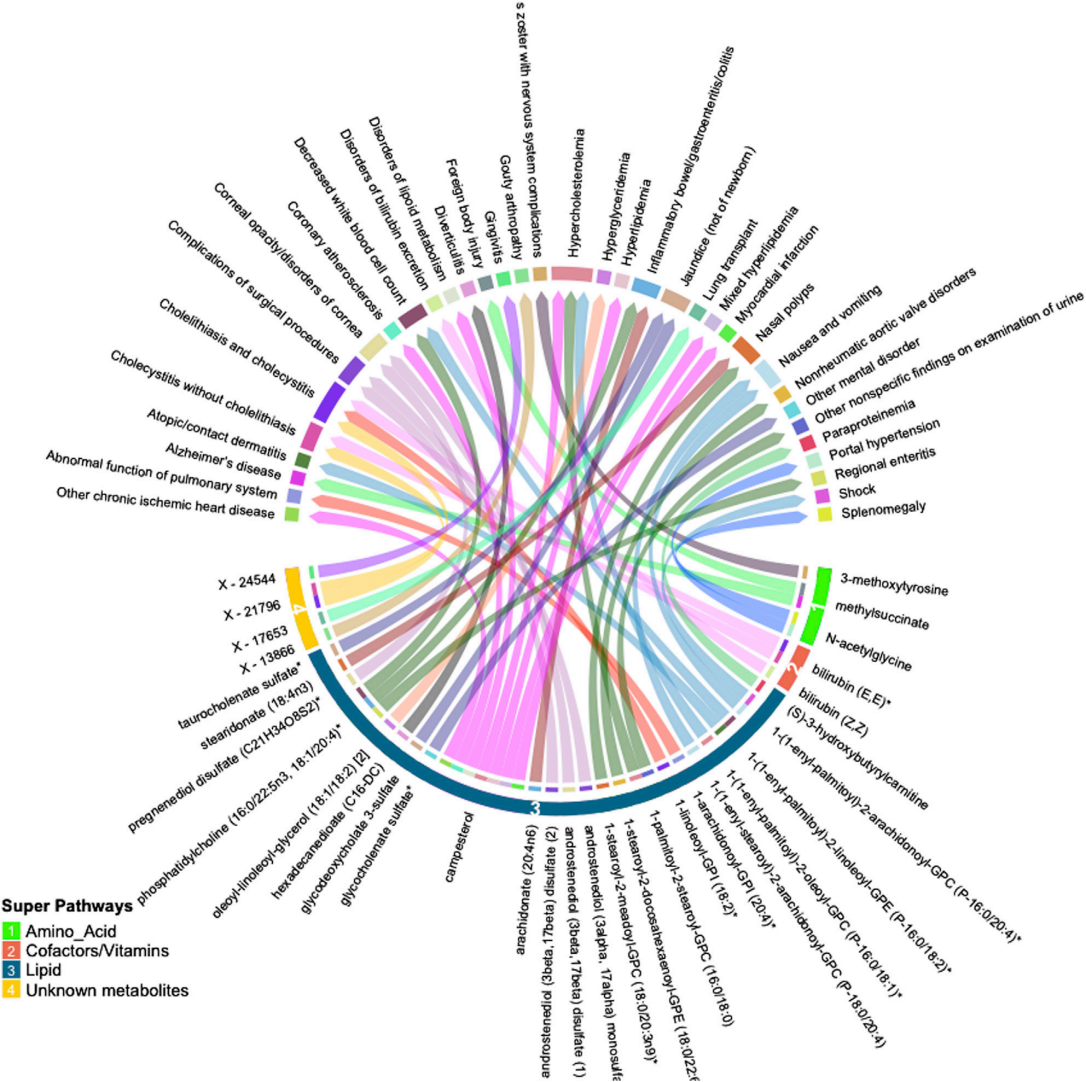
Circular plot summarizing significant associations between circulating metabolites and phenotypes identified by inverse-variance weighted (IVW) Mendelian randomization analysis (FDR *p* < 0.05). Metabolites are shown in bottom half of the figure with super-pathways depicted on the outer track (with colors and numbers) and sub-pathways shown as the color of each line (i.e., lines with the same color belong to the same sub pathway). Each color of the outer top track and the inner bottom track corresponds to a specific phenotype.

Many of these associations were driven by instruments composed of only one or two SNPs, increasing the likelihood of associations due to SNPs with pleiotropic effects. We thus selected only metabolites with genetic instruments composed of three or more independent SNPs for further validation. Similarly, to avoid spurious associations driven by pleiotropy, we excluded associations with significant heterogeneity (*p* < 0.05). After applying these exclusion criteria, 47 significant associations (FDR<0.05) among 32 metabolites and 34 phenotypes remained. A summary of the retained metabolite pairs is presented in Figure 2; Supplementary Table S3. These metabolites map to four super-pathways, with the majority mapping to lipid pathways. Distinct phenotypes with a high number of significant associations with metabolites included cholecystitis [n = 5], hypercholesterolemia [n = 3] and IBD [n = 2]. Metabolites with a high number of significant associations with phenotypes included campesterol [n = 7], phosphatidylcholine (16:0/22:5n3, 18:1/20:4) [n = 3], bilirubin (E,E) [n = 2], methylsuccinate [n = 2] and X–21,796 [n = 2].

In the African ancestry population, of 107 metabolites with significant associations in the PGS analysis, 85 had available summary statistics in the METSIM study and among unmatched metabolites, 14 were unknown. The IVW method identified 22 significant (FDR<0.05) associations comprising of 15 metabolites and 13 phenotypes (Figure 1B; Supplementary Table S4). These included several associations between lipids and infectious or acute inflammatory diseases, including urinary tract infections, sepsis, and fever.

A summary of the associations between the individual SNPs used in the genetic instrument for each metabolite and the clinical phenotypes is presented for European (Supplementary Table S5) and African (Supplementary Table S6) ancestry individuals.

### Validation of the significant association

**FIGURE 3:**
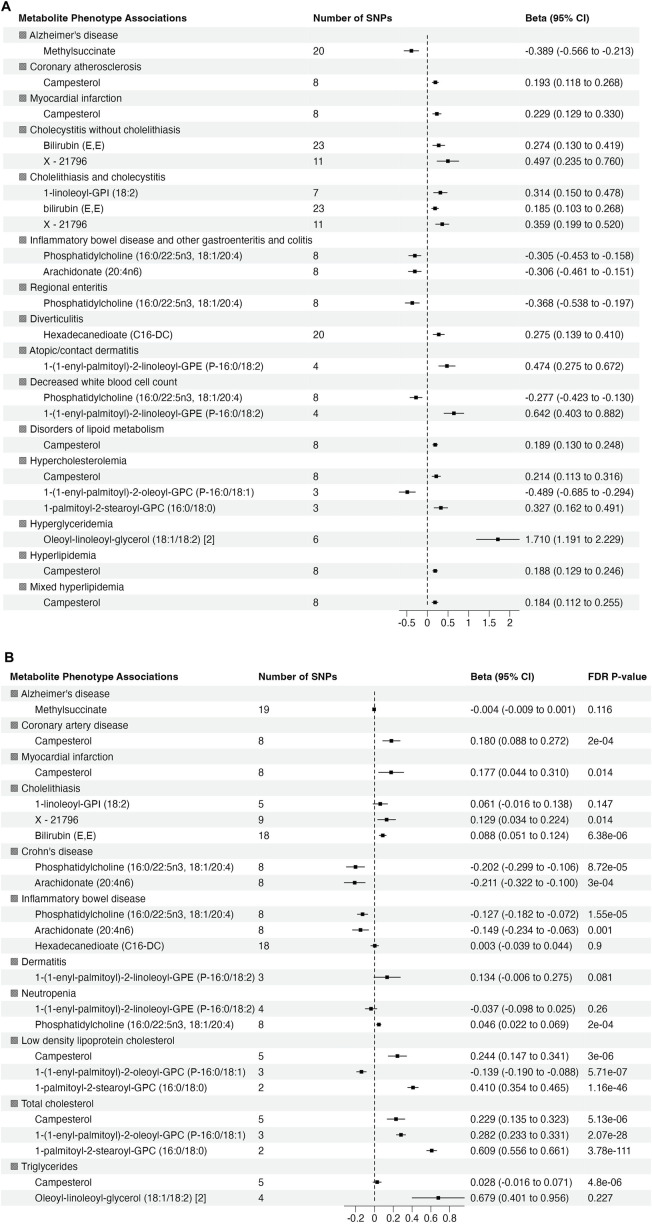
Summary of association from MR analyses between genetic instruments for metabolites in in METSIM and genetic predisposition of phenotypes derived from **(A)** BioVU (all significant at false discovery rate (FDR) *p*-value <0.05) and **(B)** validation phenotypes (The effect size and 95% confidence interval (CI) are based on raw *p*-value. However, the significant results are considered at FDR *p*-value <0.05).

To validate the significant findings from MR, we tested associations between the metabolite genetic instruments and phenotypes with available external GWAS summary statistics. After excluding associations with significant heterogeneity, <3 SNPs and non-specific phenotypes (e.g., “Other mental disorder”), there were 15 phenotypes (with 12 associated metabolites) taken forward for further validation from European ancestry (Figure 3A). There were no suitable external GWAS datasets available to evaluate the significant associations in African ancestry.

Of 22 metabolite-phenotype pairs evaluated, 16 were significant (FDR *p* < 0.05), with the same direction of effect (Figure 3B; Supplementary Table S7). Among the disease associations were bilirubin (E,E) and X–21,796 associated with cholelithiasis, phosphatidylcholine (16:0/22:5n3, 18:1/20:4) and arachidonate (20:4n6) inversely associated with inflammatory bowel disease and Crohn’s disease, and campesterol with coronary artery disease (CAD) and MI. Phosphatidylcholine (16:0/22:5n3, 18:1/20:4) was associated with low neutrophil count (neutropenia). The significant associations of phosphatidylcholine (16:0/22:5n3, 18:1/20:4) with low neutrophil count (neutropenia) and lipid diagnosis related to hypercholesteremia (total cholesterol) with 1-(1-enyl-palmitoyl)-2-oleoyl-GPC (P-16:0/18:1) were not consistent among the MR methods, suggesting that they may represent pleiotropy or are spurious.

## Discussion

Metabolites are highly relevant integrative markers of health and disease, that can inform disease prediction and pathophysiology. However, large datasets are required to robustly interrogate metabolite-phenotype associations. Measuring metabolites in large numbers of samples is costly, logistically challenging, and often unfeasible. In this “virtual” metabolomics study, we leveraged state-of-the-art genetic methods in conjunction with large, phenotypically diverse clinical and genetic data sets to interrogate the metabolome against a broad clinical phenome. Among 724 metabolites analyzed, we found 336 metabolites in European ancestry and 107 metabolites in African ancestry that showed significant associations with clinical phenotypes in the BioVU population. Of these, 159 and 22, in European and African ancestry respectively, remained significant under a MR framework. This used genetic instruments for metabolites constructed in an independent population, and consistent with the assumptions of MR, suggests they may be mediators of disease risk. Among associations identified in the European ancestry population, we independently validated associations for 16 of 22 metabolite-phenotype pairs using phenotypes derived from independent GWAS studies. Among the validated phenotypes were IBD, cholelithiasis, CAD, MI, neutropenia and lipid phenotypes. These analyses highlight the value of applying the “virtual” metabolomic approach in diverse, phenotype-rich biobanks to identify novel associations.

It is important to interpret genetically-based associations with caution, as they are susceptible to a number of biases. Some of these biases may be attenuated using two-samples approaches, as used here, which reduces spurious associations that can arise from one-sample studies (Burgess et al., 2019). Associations based polygenic predictors may be due to the effects of a single (or a small subset) of SNPs and, hence, these associations have similar limitations as epidemiological associations in that the etiological relationship between the expose and outcome is not clear (Burgess et al., 2019). Associations based on MR methods (assuming no violations of the key assumptions), can reduce the likelihood of an association driven by outlying SNPs and can provide more insights into etiological relationships (Davey Smith and Hemani, 2014). However, all associations have to be evaluated in the context of a larger and more robust knowledge base in order to further determine their validity. Hence, for several associations identified, we discuss their plausibility in the context of current clinical and experimental evidence bases.

We found consistent associations between gastrointestinal disease phenotypes and bioactive lipids, highlighting both inflammation and resolution of inflammation as important disease mediators. We found inverse associations between phosphatidylcholine (PC) (16:0/22:5n3, 18:1/20:4) and arachidonate (20:4n6) with IBD and Crohn’s disease, both inflammatory diseases of the gut mucosa (Alhouayek et al., 2021). Circulating phosphatidylcholines have been reported to be reduced in inflammatory bowel disease, suggesting that they may have a protective role in the gut mucosa (Treede et al., 2007; Stremmel et al., 2021). PCs may have anti-inflammatory effects and prevent mucosal damage (Treede et al., 2007), with potential therapeutic application for IBD (Ai et al., 2022). It is important to identify the specific PC involved in protecting the gut against disease. One of the abundant main species of phosphatidylcholines in gut mucus is PC 16:0/18:1 (Treede et al., 2007). This is consistent with our data indicating that lower genetically-predicted phosphatidylcholine (16:0/22:5n3, 18:1/20:4) associates with IBD and Crohn’s disease. The association between arachidonate (20:4n6) with IBD and Crohn’s disease may have been biased due to unaccounted pleiotropy; however, as MR-PRESSO can correct for the contributions of outlying SNPs, the corrected estimate provided by MR-PRESSO should be considered as a more reliable estimate (Zhu, 2021). There is biological support for the association between arachidonate (20:4n6) and IBD in the literature. Arachidonic acid is a precursor of eicosanoids, with potential anti-inflammatory activity (Marton et al., 2019), and has previously been shown to be inversely associated with IBD including UC and Crohn’s disease (Levy et al., 2000; ROMANATO et al., 2009; Bugajska et al., 2022).

We observed several other plausible disease specific associations. There were positive associations between both bilirubin (E,E) and X–21796 with cholelithiasis (gallstone disease). A causal association has previously been reported between extreme levels of bilirubin and increased risk of gallstone disease (Stender et al., 2013). Bilirubin (E,E) is one of the water soluble isomers of bilirubin that is converted from unconjugated bilirubin (Z,Z) upon exposure to light (Wang et al., 2021). The identity of X–21796 is unknown. However, SNPs associated with X–21796 map to several members of the *UGT1A* family of genes, which have also been associated with bilirubin levels and risk of gallstones (Stender et al., 2013), and *SLCO1B,* which is involved in bilirubin transport into the liver (Keppler, 2014). This suggests that this unknown metabolite may be closely related to bilirubin, and also highlights the utility of our approach to define the underlying mechanistic basis of associations with unknown metabolites using genetic data, which is generally not feasible using other standard epidemiological approaches.

Interestingly, the “virtual” metabolomics approach provided us with a considerable opportunity for novel discovery in relation to cardiovascular disease (CVD). Previously, a meta-analysis found no association between serum concentrations of two common plant sterols (sitosterol and campesterol) and risk of CVD (Genser et al., 2012). However, in our large well-powered study, we found a positive association between campesterol and risk of CAD and MI. Campesterol was also strongly associated with most of the phenotypes categorized in the lipid-related disorders group. Several factors have been proposed as the potential mechanisms linking elevated concentration of campesterol and increased CVD risk, including common pathways influencing the absorption of cholesterol and plant sterols in the intestines, (Silbernagel et al., 2010), shared genetics linking lipoproteins and phytosterols to MI and atherosclerosis, (Wang et al., 2014; Scholz et al., 2022), poor nutritional status, (Strandberg and Pitkälä, 2007), and poor metabolic health (Simonen et al., 2000). We anticipate that future analyses may validate and explore the mechanistic bases and underlying pathophysiology of this interesting finding.

This unbiased discovery approach allowed us to create and validate a resource of associations which identified metabolites that are biomarkers and potential mediators of several other clinical phenotypes. For instance, we successfully validated an inverse association between the plasmalogen 1-(1-enyl-palmitoyl)-2-oleoyl-GPC (P-16:0/18:1) and hypercholesterolemia. This metabolite was reported as inversely related to visceral adipose tissue volume and the percentage of fat in the liver and pancreas (Lind et al., 2021). We also found associations between 1-palmitoyl-2-stearoyl-GPC (16:0/18:0) and LDL and total cholesterol; this metabolite has been found to be positively associated with dyslipidemia (Yousri et al., 2022). Our data demonstrated that hypertriglyceridemia was positively associated with oleoyl-linoleoyl-glycerol (18:1/18:2), potentially a novel association. We also found and validated a significant association between phosphatidylcholine (16:0/22:5n3, 18:1/20:4) and low blood cell count (neutropenia). There were other interesting associations we were unable to validate using external data sets due to lack of available data. For instance, we observed positive significant associations between stearidonate (18:4n3) and 1-stearoyl-2-meadoyl-GPC (18:0/20:3n9) and nasal polyps. Dysregulated lipid metabolism has been reported in nasal polyps (Miyata et al., 2019). These metabolites potentially represent new biomarkers of this disorder. An inverse association between methylsuccinate and Alzheimer’s disease (AD) was not validated, however given published data linking methylsuccinate supplementation to improvement in neuron dysfunction in AD, this may merit further study.

A significant strength of this study was the use of large datasets which have proven robust for discovery of SNPs associated with both metabolites and disease. A further strength is that we utilized genetic approaches that are well-validated for the applications we propose (Voight et al., 2012; Larsson et al., 2017). We analyzed data from multiple sources, including multiple non-overlapping independent cohorts using independent metabolite measurement platforms, and analysis in both European and African ancestry populations where possible. This allowed us to maximize discovery through increased sample sizes and a more diverse population sample, to ensure generalizability, reproducibility and rigor of the association (Vsevolozhskaya et al., 2017). Moreover, validating the observed associations using available external GWAS additionally strengthened our findings.

Our study also has some limitations. An important limitation of a genetics-based association approach is that the association may not be consistent when using directly measured levels of the metabolite. This can be due to pleiotropic associations, such as when a SNP in the predictor tags a genetic locus that is associated with an outcome through a mechanism unrelated to the metabolite, or due to weak instrument bias (Davies et al., 2015; Gianola et al., 2015). When selecting genetic instruments for metabolites in the MR studies, was also used a permissive inclusion threshold (an association p < 5 × 10^−6^), which can drive weak instrument bias. Further, some metabolites are heavily modulated by environment and homeostatic physiology, which may mask an association. Metabolites are also highly inter-correlated, which was confirmed by a high level of overlap within SNP predictors. We elected to treat each metabolite individually, as is standard for metabolomic association studies, without accounting for the correlation structure. This may have resulted in overly-stringent correction for multiple testing. Future focused studies are required to investigate the relationships between combinations of metabolites and disease. GWAS data were not available for all the phenotypes showing a significant association with metabolites. This limited the number of total novel findings we could evaluate in external data sets. We were also limited in our ability to detect ancestry-specific effects. The metabolite predictors were constructed in European (OMICSPRED) and Finnish (METSIM) ancestry individuals. Thus, these may not be appropriate instruments for identifying metabolite-disease associations in individuals of African ancestry.

In summary, we identified novel metabolite-phenotype associations, and confirmed known relationships between metabolites and disease. Further studies are needed to replicate and clinically validate these findings. This study highlights the utility of a genetics-based “virtual” metabolomics approach in conjunction with DNA biobanks to link metabolites to clinical diseases and clinical diagnoses. As genetic biobanks continue to grow, the potential to discover genetic underpinnings of the metabolome will also expand. This approach can be used to identify additional metabolite-disease associations, uncover novel disease biology and move towards application in clinical populations.

## Data Availability

The data analyzed in this study is subject to the following licenses/restrictions: The complete summary results of all analyses are presented in the Supplementary Material. Subject-level access to BioVU clinical and genetic data is controlled by the BioVU data repository (https://victr.vumc.org/biovu-description/#). Upon publication, data to replicate the primary findings presented here for research purposes may be requested from the repository (biovu@vumc.org). BioVU vetting for use of individual-level data includes institutional IRB approval, data use agreements, and administrative and scientific reviews. Requests to access these datasets should be directed to biovu@vumc.org.
